# Supplementary material for: The Evolution and Characterization of the RNA Interference Pathways in Lophotrochozoa
Source: Genome Biol Evol. 2024 May 7;16(5):evae098. doi: 10.1093/gbe/evae098 (PMC11114477; doi:10.1093/gbe/evae098)
Supplement: evae098_Supplementary_Data [file evae098_supplementary_data.zip › Supplementary_materials.zip!/selected_miRNAs/NoGe_selected_miRNAs/known_miRNA_families_pdf_files/NMRB01000212.1_Notospermus_geniculatus_strain_Ushimado_scaffold212,_whole_genome_shotgun_sequence_7288.pdf]

| | | |
|---|---|---|
| Provisional ID | : | NMRB01000212.1_Notospermus_geniculatus_strain_Ushimado_scaffold212,_whole_genome_shotgun_sequence_7288 |
| Score total | : | 930.5 |
| Score for star read(s) | : | 3.9 |
| Score for read counts | : | 921.9 |
| Score for mfe | : | 1.7 |
| Score for randfold | : | 0 |
| Score for cons. seed | : | 3 |
| Total read count | : | 1820 |
| Mature read count | : | 1813 |
| Loop read count | : | 0 |
| Star read count | : | 7 |

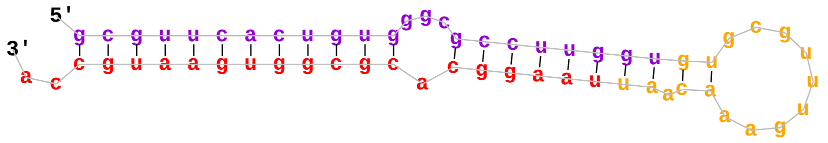

Genome coordinates: NMRB01000212.1 41950-42010

pre-miRNA sequence:
GCGUUCACUGUGGGCGCCUUGGUGCGUUCACUGUGGGCGCCUUGGUGUGCGUUUGAAACAAUUAAGGCACGCGGUGAAUGCCA

Mature sequence: UAAGGCACGCGGUGAAUGCCA

Aligned mature sequence of the *N. geniculatus* pre-miRNA with the top 5 hits from the BLAST search against the pre-miRNA mirGeneDB set:

```
                                         *********************
    nge_Mir-124                         -uaaggcacgcggugaaugcca-
    Xtr-Mir-124-P1-v2_3p                uuaaggcacgcggugaaugcca-
    Xtr-Mir-124-P2-v1_3p                -uaaggcacgcggugaaugccaa
    Xtr-Mir-124-P2-v2_3p                uuaaggcacgcggugaaugcca-
    Xtr-Mir-124-P3-v1_3p                -uaaggcacgcggugaaugccaa
    Xtr-Mir-124-P3-v2_3p                uuaaggcacgcggugaaugcca-
```
